# Duodenal Diverticulitis Following Biliopancreatic Diversion: A Case Report

**DOI:** 10.7759/cureus.45219

**Published:** 2023-09-14

**Authors:** Timothy W Ritchie, Zachary M Wargel, Emanuel Shapera, Andrew A Wheeler

**Affiliations:** 1 Surgery, University of Missouri School of Medicine, Columbia, USA; 2 General Surgery, Grossmont Surgical Associates, San Diego, USA

**Keywords:** complications, bariatric surgery, transgastric endoscopy, biliopancreatic diversion, duodenal diverticulitis

## Abstract

Duodenal diverticulitis is a relatively uncommon finding in patients. Treatment of complications of duodenal diverticulitis may be challenging in patients with altered intestinal anatomy such as those with altered anatomy from weight loss procedures involving intestinal bypass. We present a case report describing the management of duodenal diverticulitis following a biliopancreatic diversion, our decision-making process, and our final treatment strategy.

## Introduction

Small bowel diverticulosis is uncommon, with the duodenum being the most common site of small bowel diverticulosis [1]. Typically, they are considered false diverticulum as only the mucosa protrudes through the muscularis propria [2]. Depending on the location of the duodenal diverticulum, these diverticula can cause biliary duct obstruction [3] or bleeding [4] secondary to inflammation related to diverticulitis. These complications are rare, occurring in 10% of cases of duodenal diverticulum [5-7]. Patients who have had prior weight loss surgery with bypassed duodenum are not immune to having duodenal diverticulum and the subsequent complications. However, these complications may present diagnostic and management challenges because of altered intestinal anatomy. We present a case report of the only reported case of hemorrhagic duodenal diverticulitis in a patient with prior intestinal bypass for weight loss and an effective treatment strategy for this challenging scenario where the duodenum cannot be directly accessed endoscopically.

## Case presentation

**Figure 1:**
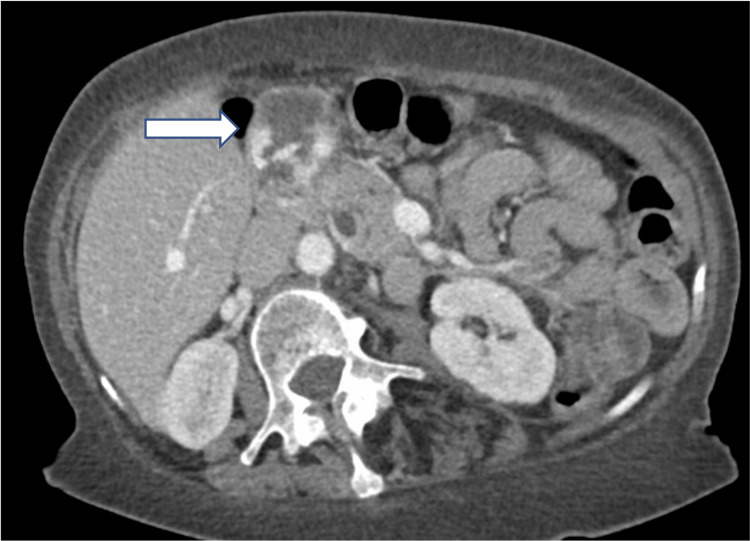
CT scan of abdomen showing duodenal diverticulum (arrow). On initial review by surgeons and radiologists the CT images were concerning for duodenal perforation with abscess.

A 64-year-old female presented with a two-week history of abdominal pain, emesis, and oral intolerance. Her past medical history was remarkable for a biliopancreatic diversion procedure performed 30 years previously. Further evaluation demonstrated chronic anemia, which was significant enough to require prior blood transfusions. A computed tomography (CT) scan of the abdomen and pelvis with IV contrast was performed to evaluate her abdominal pain, and the CT demonstrated findings concerning a contained duodenal perforation (Figure 1). Based on these findings, she was taken to the operating room for emergent diagnostic laparoscopy.

**Figure 2:**
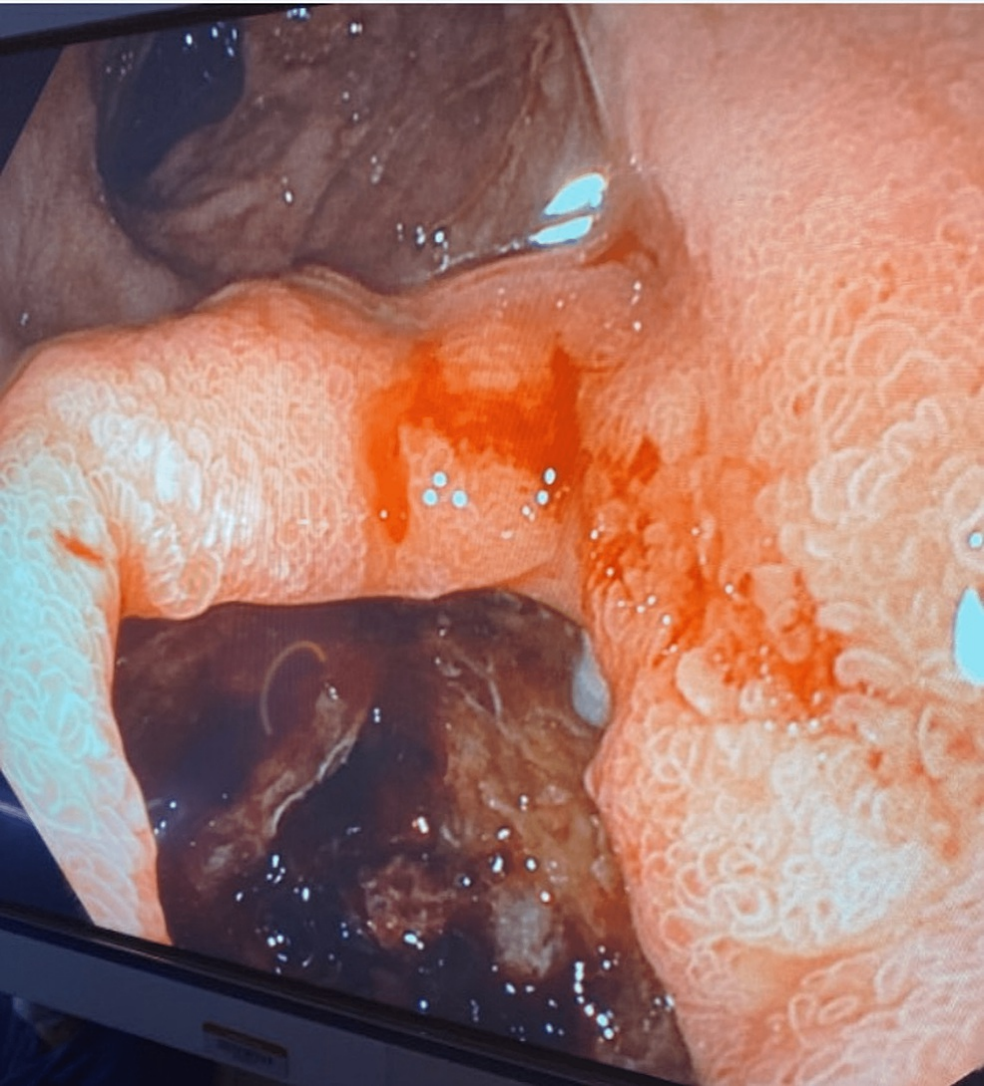
Duodenal diverticulum with hemorrhage. Duodenal diverticulum located within the second portion of the duodenum. Diverticulum on bottom of the picture with a more distal duodenum on top of the figure.

An upper endoscopy was first performed revealing a non-bleeding ulcer at the gastrojejunal anastomosis. A diagnostic laparoscopy failed to review any intraabdominal pathology including the absence of duodenal perforation. Given the CT findings and absence of intraabdominal findings during laparoscopy, the decision was made to perform a transgastric gastroduodenoscopy. The greater curvature of the stomach was secured with 2-0 silk stay sutures and a bladed 12 mm trocar was inserted through the abdominal wall into the stomach. A gastroscope was inserted through the trocar into the stomach and then advanced into the duodenum. The remnant stomach was normal, but upon entering the second portion of the duodenum, a diverticulum with a 3-cm ulcer was seen approximately 2 cm proximal to the ampulla of Vater (Figure 2). Hematoma was present in the inflamed diverticulum consistent with hemorrhagic diverticulitis. The blood was evacuated, and no visible vessel was present. A nodular area was biopsied, returning several days later as benign mucosa. The transgastric trocar was removed and replaced with a 24 French gastrostomy tube in a standard Stamm fashion to provide future access to the stomach in the event that future access to bypass the stomach and intestine is required. The stomach was sutured to the abdominal wall with permanent sutures to prevent gastric leakage around the tube. The patient convalesced well for the first three days.

On postoperative day four, the patient was observed to have dark sanguineous output from her gastrostomy tube with reported dark stools. Hemoglobin decreased, requiring repeat blood transfusion. Initially, the hemoglobin stabilized but decreased two days later from 8.7 to 7.7 grams/deciliter. Thus, the decision was made to perform a repeat upper endoscopy in a transgastric manner through the gastrostomy tube site left during diagnostic laparoscopy. An esophagogastrojejunoscopy was also performed with a bleeding marginal ulcer identified and treated endoscopically with argon plasma coagulation. A gastroscope was then passed through the gastrostomy tube site to access the remnant stomach and duodenum for inspection of the previously identified hemorrhagic duodenal diverticulitis. A small amount of blood was evacuated from the duodenal diverticulum, but no active bleeding or visible vessels were found. The gastroscope was removed, and the gastrostomy tube was replaced. The patient recovered well postoperatively. On postoperative day two of the patient’s second operation, she was discharged on a 14-day total course of ciprofloxacin and metronidazole for duodenal diverticulitis and a proton pump inhibitor. One month later at outpatient follow-up, the patient was doing well with no recurrence of hemorrhage from the duodenum with stable hemoglobin levels, so the gastrostomy tube was removed.

## Discussion

After the colon, the duodenum is the most common location for intestinal diverticula [8,9] and the most common site for small bowel diverticulosis [1]. Even so, they are uncommon, occurring in 0.2%-4.5% of patients in autopsy studies and diagnosed in 0.5%-2.3% of living patients. Diverticula are characterized as “true” when they compose all layers of the intestine and “false” when only the mucosal or submucosal layers are involved [8,10]. Small bowel diverticula may also be classified based on location (intraluminal or extraluminal) and etiology (congenital or acquired), the latter of both characteristics being the most common [7]. They occur more frequently in patients above the age of 40 as intestinal muscle integrity deteriorates [5,8,10]. Most cases are discovered incidentally, with only 5-10% of patients presenting symptomatically [5,8,11]. Common complications of duodenal diverticulum include cholestasis, inflammation, hemorrhage, abscess, intestinal obstruction, and perforation [8,9,12]. A weakened intestinal wall coupled with increases in intraluminal pressure are the suspected causes of diverticula formation; hence, they tend to occur near the ampulla of Vater or near blood vessels that course in and out of the intestinal wall. When these diverticula become obstructed, they can cause inflammation leading to diverticulitis. Inflammation eroding into intramural blood vessels can result in hemorrhage into the diverticulum. Obstructive jaundice can rarely occur from diverticulum, a condition known as Lemmel’s syndrome [3,13]. Additionally, like diverticulum in other parts of the gastrointestinal tract, a perforation can occur leading to intraabdominal abscess or sepsis [14]. Patients with duodenal diverticulum may have a genetic predisposition, with obesity increasing the risk for acquired diverticular disease [15]. Given that patients undergoing weight loss surgery have obesity to begin with, they are at higher risk of acquiring small bowel diverticulosis. 

A duodenal diverticulum can be more difficult to assess and treat in bariatric surgery patients who have undergone intestinal bypass leading to exclusion of the duodenum. Thus, when complications arise such as hemorrhage or acute diverticulitis, an awareness of this pathology is important because of diagnostic limitations. CT scanning has proven useful in detecting complications associated with duodenal diverticulitis, and with recent technological advances, has improved the diagnosis of mall bowel diverticular disease [16]. They are less useful in the identification of duodenal diverticular bleeding in the bypassed intestine as a cause of acute or chronic blood loss anemia. When hemorrhage is suspected, diagnostic endoscopy with gastroscopy through the bypassed stomach is often necessary. The duodenal diverticulum is hard to visualize in locations such as at the mesenteric border or between the leaves of the mesentery, increasing the risk of misdiagnosis even during a diagnostic laparoscopy. The inability to utilize oral endoscopy to reach the bypassed duodenum compounds the difficulty in diagnosing abnormal pathology. Needless to say, the operating surgeon must have familiarity and experience in treating patients with altered anatomy and utilize transgastric endoscopy to evaluate the remnant stomach and duodenum when other first-line modalities, such as diagnostic laparoscopy, oral endoscopy for marginal ulcer, or colonoscopy for colonic bleeding, fail to identify the source of blood loss.

For patients who develop duodenal diverticulitis, conservative treatment in stable patients includes empiric intravenous antibiotics along with dietary modifications [11,12]. If ineffective, surgical management may be indicated, although it is often reserved for instances of diverticular perforation [12]. For those with hemorrhage complicating duodenal diverticulitis, endoscopic options are often utilized [13]. When presented with a bypassed duodenum such as in our patient, a transgastric approach is needed for endoscopic hemorrhage control.

To the author's knowledge, there have been only two other cases reported of small bowel diverticulitis following altered gastrointestinal anatomy secondary to intestinal bypass procedures for obesity [6,15]. Both cases were after gastric bypass with described diverticula formation in the Roux limb, which was accessible by upper endoscopy; even so, both patients required resection and anastomosis of the Roux limb. This patient presented a greater diagnostic challenge as the diverticulum was in the bypassed duodenum after a biliopancreatic diversion procedure. She had suffered from chronic anemia and malnutrition, likely caused by a combination of duodenal diverticulitis and the intestinal bypass procedure. This patient required surgical access via the remnant stomach to access the duodenum to allow for endoscopic diagnosis and treatment. The CT scan initially seemed to indicate a contained duodenal perforation, but., during diagnostic laparoscopy, no perforation was identified. Based on the absence of intraoperative findings, intraluminal pathology was suspected. To obtain a definitive diagnosis, access to the remnant stomach and proximal duodenum must be achieved as presented. This can be done effectively through the placement of a 12- or 15-mm trocar through the abdominal wall and then into the remnant stomach using stay sutures to provide countertraction. The operative field can then be draped and protected, allowing the non-sterile gastroscope to access the remnant stomach without compromising the sterile operative field. Gastroduodenoscopy can then be performed in a transgastric manner to evaluate the remnant stomach and duodenum. When combined with esophagogastrojejunoscopy, the entire upper gastrointestinal tract can be evaluated to the level of the ligament of Treitz. This case highlights the utility of laparoscopic gastric remnant access to facilitate the diagnosis and treatment of duodenal diverticula or other duodenal/remnant stomach pathology in patients with intestinal bypass anatomy. This is a technique that bariatric surgeons caring for complex patients should have in their armamentarium. 

## Conclusions

Gastric bypass and biliopancreatic diversion are effective surgical options for patients with morbid obesity. The altered anatomy limits endoscopic access to the hepatobiliary system and duodenum, which can complicate disease treatment in these locations. Adjunctive laparoscopic gastric remnant access facilitating gastroduodenoscopy to manage duodenal diverticular disease is an effective and safe treatment option for this rare disease.
